# Persistence versus dynamical seasonal forecasts of cereal crop yields

**DOI:** 10.1038/s41598-022-11228-2

**Published:** 2022-05-06

**Authors:** Virgílio A. Bento, Ana Russo, Emanuel Dutra, Andreia F. S. Ribeiro, Célia M. Gouveia, Ricardo M. Trigo

**Affiliations:** 1 Instituto Dom Luiz, Faculdade de Ciências da Universidade de Lisboa, 1749 – 016 Lisboa, Portugal; 2 Instituto Português Do Mar E da Atmosfera, I.P., Rua C do Aeroporto, 1749 – 077 Lisboa, Portugal; 3 Institute for Atmospheric and Climate Science, ETH Zurich, Universitätstrasse 16, 8092 Zurich, Switzerland

**Keywords:** Atmospheric science, Environmental impact

## Abstract

Climate change is expected to have impacts on the balance of global food trade networks and food security. Thus, seasonal forecasts of precipitation and temperature are an essential tool for stakeholders to make timely choices regarding the strategies required to maximize their expected cereal yield outcomes. The availability of state-of-the-art seasonal forecasts such as the European Centre for Medium-Range Weather Forecasts (ECMWF) system 5 (SEAS5) may be an asset to help decision making. However, uncertainties and reduced skill may hamper the use of seasonal forecasts in several applications. Hence, in this work, we aim to understand the added value of such dynamical forecasts when compared to persistent anomalies of climate conditions used to predict the production of wheat and barley yields. With that in mind, empirical models relating annual wheat and barley yields in Spain to monthly values of precipitation and temperature are developed by taking advantage of ECMWF ERA5 reanalysis. Then, dynamical and persistence forecasts are issued at different lead times, and the skill of the subsequent forecasted yield is verified through probabilistic metrics. The results presented in this study demonstrate two different outcomes: (1) wheat and barley yield anomaly forecasts (dynamical and persistent) start to gain skill later in the season (typically from April onwards); and (2) the added value of using the SEAS5 forecast as an alternative to persistence ranges from 6 to 16%, with better results in the southern Spanish regions.

## Introduction

Wheat and barley crops are paramount to food supply chains at global and national levels. Indeed, these cereals are part of the diet and the nutritional basis of humans and animals^1^. Rainfed winter wheat and barley are vital crops in Spain, representing approximately 23 and 31% of the total crop area in Spain in 2019, i.e., more than half of the total cereal cultivation area of the country (https://www.mapa.gob.es). Furthermore, rainfed winter wheat and barley in Spain represent about 8 and 25%, respectively, of the total EU-27 areas dedicated to these crops, being the main producer of barley and the fifth largest producer of wheat in the EU-27 (EUROSTAT, 2020). Thus, the importance of steady and consistent production of these cereals every year is of critical importance to stakeholders (such as farmers), countries’ economies, and the European supply of wheat and barley. Recent studies show how climate-related impacts on European crop production have tripled over the last five decades^2^, representing a present risk for food security. Increasing crop shortfalls induced by weather disasters are also expected at the global level^3,4^. If poor harvest occurs across multiple regions simultaneously, the global food system may be heavily compromised^5,6^, particularly in a changing climate.

To make farsighted strategic choices, stakeholders need to have timely, accurate, and reliable knowledge of crop yield production at their disposal^7^. Considering the importance of meteorological conditions to cereal production, seasonal forecasts of meteorologic variables such as precipitation and temperature have a key role^8^. Several works have studied the use of different seasonal forecasts on crop yields located in different regions of the world^9–16^ and have adopted different methodologies to estimate crop yields, ranging from crop models forced with seasonal forecasts^17^ to empirical models based on simple regressions^15,18^ or more advanced statistical methods such as machine learning^19^.

Since 2017, the latest generation of the European Centre for Medium-Range Weather Forecasts (ECMWF) seasonal forecasting system version 5 (SEAS5) has become operational, providing an ensemble of the evolution of the coupled earth system (atmosphere, land, ocean) with lead times up to 6 months. Such seasonal forecasts could be an essential tool for stakeholders to make timely, conscious, and proactive choices regarding the actions necessary to maximize the production of wheat and barley yields by choosing ideal management strategies. However, the quality of dynamic forecasts pose a significant hurdle for farmers and other stakeholders to use them^20^. Indeed, forecasts with longer lead times are generally less accurate than those with shorter ones, and precipitation forecasts are less accurate than temperature forecasts^20^. Hence, it is crucial to evaluate the added value of such dynamical forecasts when compared with simpler approaches, such as assuming forecasts based on persistent climate conditions, i.e., assuming that anomalies persist in the following months. The aim of this study is to compare cereal yield production (specifically wheat and barley in Spain) using linear regression models fed with dynamical and persistence forecasts, and to compare the skill achieved by both. To accomplish this, the following two steps are proposed:

1. *Model Building*: To select and calibrate linear regression models to estimate wheat and barley by using ERA5 monthly accumulated precipitation and minimum and maximum temperatures over the cereals’ growing season. This first step is based on prior publications by the authors and is only adapted here to the proposed database of ERA5^18,21^.
2. *Forecast*: Dynamic (SEAS5) and persistency forecasts of these variables are fed into the linear regression models to obtain forecasted wheat and barley yield estimates. The skill of both forecasts of these yields was compared, taking the yield estimated with ERA5 as the “benchmark”. This aims to understand if using seasonal forecasts of the predictors is a better strategy than relying on the persistent climate (which relies on the assumption that the climate anomaly of the previous month will be the climate anomaly of the next months). This second step, which constitutes the main goal of this study, aims at analysing the added value of using dynamical seasonal forecasts to predict cereal yields and informing end-users (e.g., farmer) and forecast developers on the usefulness of such datasets in the regions of study for the cereals in question.

## Data and methods

### Region of study and agriculture data

**Figure 1:**
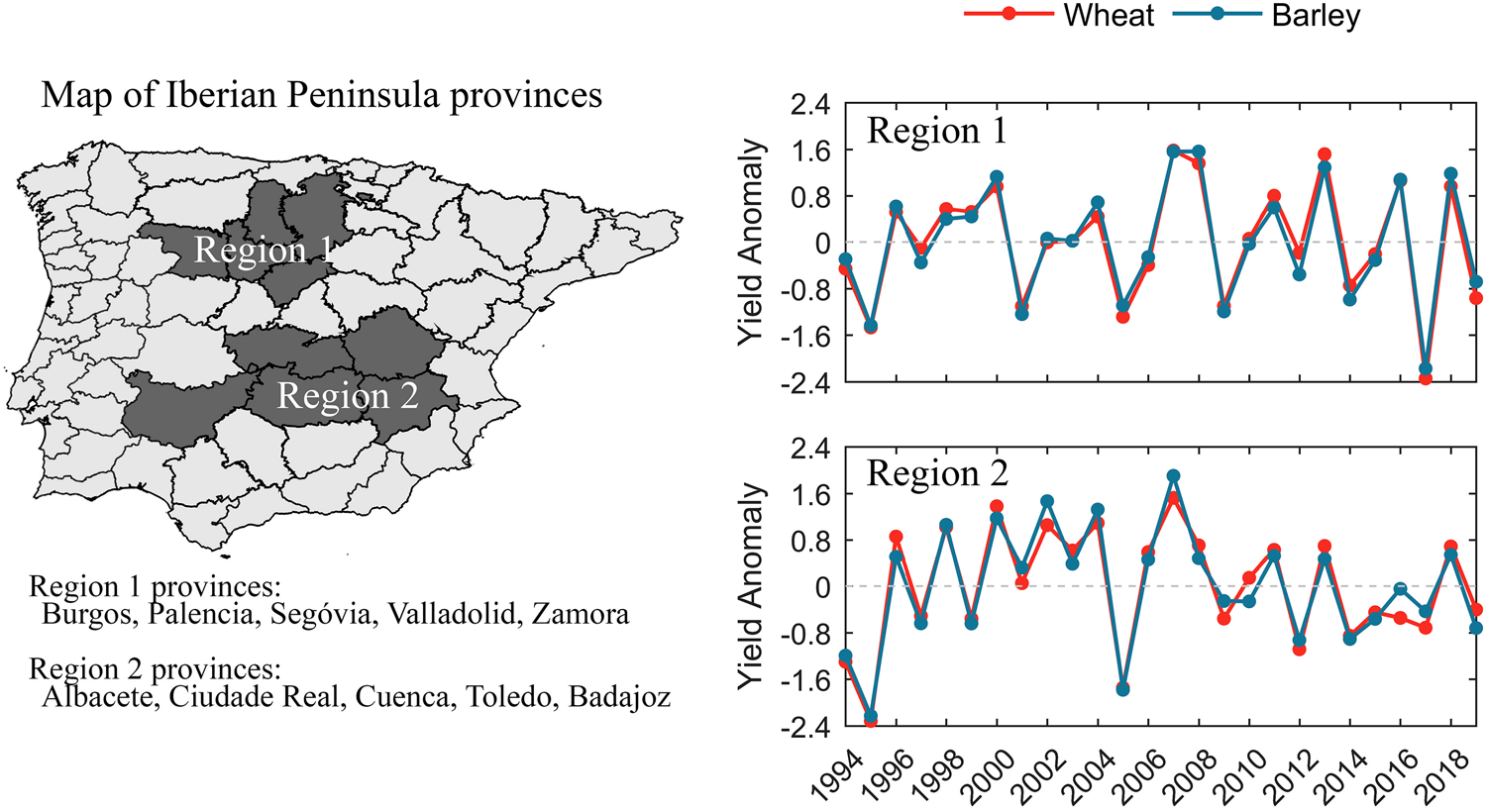
Map highlighting selected adjacent provinces in the Iberian Peninsula, called Region 1 and Region 2 (left), and the respective province names and time series of wheat (orange) and barley (blue) standardized yield anomalies for the period 1993–2019 for Region 1 (right, top panel) and for Region 2 (right, bottom panel). The figure was produced in Python 3.8 (https://www.python.org/).

Two different regions located in Spain, encompassing five adjacent provinces each, are chosen as the focus of this study. These provinces were selected because of their large percentages of agricultural land use dominated by rainfed crops, such as wheat and barley (Fig. 1, left panel). The choice of such a collection of provinces is built upon previous works^18,21,22^, where similar crop systems and regions were analysed. The main rationale for the choice of the provinces builds upon three criteria^18^: (1) the land use within the province is dominated by agricultural practices, i.e., more than 50% of the pixels at each province correspond to agricultural areas; (2) the agricultural areas are dominated by rainfed crops, i.e., non- irrigated arable land prevails in more than 50% of the agricultural areas; (3) the provinces are adjacent and non-isolated.

Wheat and barley annual production (ton) and agriculture-specific area (ha) for each of the selected provinces were obtained from the Spanish Agriculture, Food and Environment Ministry (available at https://www.mapa.gob.es). Average production of wheat and barley is of 2.9 and 2.8 ton/ha, respectively, in region 1, and 2.3 ton/ha in region 2, for both cereals. Annual yield anomalies (ton/ha) were then estimated for each individual province (dividing the yearly production of cereals in ton by the area assigned to that crop in ha in the respective province), and the regions’ spatial average was finally obtained. Furthermore, long-term trends associated with nonclimatic factors such as technological progress were removed by linear detrending^23,24^. Finally, the time series were standardized (Fig. 1, right panel). To simplify notation, let us consider the northernmost region as Region 1 (shown in blue in Fig. 1) and the central/southern Spanish region as Region 2 (orange in Fig. 1).

Annual wheat and barley production datasets are available since 1993 (see subsection 2.3) until the last available report pertaining to 2019.

### Precipitation and temperature reanalysis

ERA5^25^ hourly fields of precipitation and 2-m temperature were retrieved from the Copernicus Climate Data Store. These were subsequently converted to (1) monthly accumulated precipitation (hereafter designated by PR_ERA5_, in mm) by accumulating the hourly fields; (2) monthly maximum temperature (hereafter TX_ERA5_, in K) obtained as the monthly mean of daily maximum 2-m temperature; and (3) monthly minimum temperature (hereafter TN_ERA5_, in K) following a similar rationale as in TX_ERA5_ but using daily minimum 2-m temperature.

ERA5 fields are available from 1950 onwards. However, data are truncated to encompass the period starting in January 1993 (see subsection 2.3) and ending in December 2019 (see subsection 2.1). Precipitation and temperature fields were spatially aggregated for each region.

### Precipitation and temperature seasonal forecasts

The ECMWF seasonal forecast system SEAS5^26^ is used in this study. Monthly reforecasts (hereafter hindcasts) of total precipitation (m s^−1^) and maximum and minimum 2-m temperatures (K; hereafter TX_S5_ and TN_S5_, respectively) at a spatial resolution of 1° × 1° are available from 1993 until 2016, whereas forecasts are employed from 2017 to 2019. Both hindcasts and forecasts start on the 1st of each month, with a 6-month lead time, and are composed of 25 ensemble members. In this study, a lead-time of 0 indicates the forecast for the issue month (e.g., for the forecasts issued in January, lead-time 0 is respective to the January forecast, and lead-time 5 to the June forecast).

Total precipitation is converted from monthly means to monthly accumulated values by multiplying by the number of days in the month and is converted to mm, hereafter referred to as PR_S5_. Furthermore, the ensemble mean of the PR_S5_, TX_S5_ and TN_S5_ fields is obtained by applying the arithmetic mean over the 25 members.

Coupled atmosphere–ocean models have systematic biases and drift (lead time-dependent biases)^27^. To guarantee a similar mean climate between SEAS5 and ERA5, a simple bias correction was applied in the following form:

1 $$ {\text{PR}}_{{{\text{S}}5}}^{^{\prime}} \left( {{\text{m}},{\text{lt}}} \right) = {\upalpha }_{{{\text{PR}}}} \left( {{\text{m}},{\text{lt}}} \right) \times {\text{PR}}_{{{\text{S}}5}} \left( {{\text{m}},{\text{lt}}} \right) $$

2 $$ {\text{TX}}_{{{\text{S}}5}}^{^{\prime}} \left( {{\text{m}},{\text{lt}}} \right) = {\upalpha }_{{{\text{TX}}}} \left( {{\text{m}},{\text{lt}}} \right) + {\text{TX}}_{{{\text{S}}5}} \left( {{\text{m}},{\text{lt}}} \right) $$

3 $$ {\text{TN}}_{{{\text{S}}5}}^{^{\prime}} \left( {{\text{m}},{\text{lt}}} \right) = {\upalpha }_{{{\text{TN}}}} \left( {{\text{m}},{\text{lt}}} \right) + {\text{TN}}_{{{\text{S}}5}} \left( {{\text{m}},{\text{lt}}} \right) $$

where ${\text{PR}}_{{{\text{S}}5}}^{^{\prime}}$, ${\text{TX}}_{{{\text{S}}5}}^{^{\prime}}$, and ${\text{TN}}_{{{\text{S}}5}}^{^{\prime}}$ are the corrected seasonal forecasts; ${\upalpha }$ is the correction factor for each of the three variables; and ${\text{m}}$ and ${\text{lt}}$ are the calendar month and lead time, respectively. The correction factors are estimated as:

4 $$ {\upalpha }_{{{\text{PR}}}} \left( {{\text{m}},{\text{lt}}} \right) = \frac{{\overline{{{\text{PR}}}}_{{{\text{ERA}}5}} \left( {{\text{m}}_{{\text{v}}} } \right)}}{{\overline{{{\text{PR}}}}_{{{\text{S}}5}} \left( {{\text{m}},{\text{lt}}} \right)}} $$

5 $$ {\upalpha }_{{{\text{TX}}}} \left( {{\text{m}},{\text{lt}}} \right) = \overline{{{\text{TX}}}}_{{{\text{ERA}}5}} \left( {{\text{m}}_{{\text{v}}} } \right) - \overline{{{\text{TX}}}}_{{{\text{S}}5}} \left( {{\text{m}},{\text{lt}}} \right) $$

6 $$ {\upalpha }_{{{\text{TN}}}} \left( {{\text{m}},{\text{lt}}} \right) = \overline{{{\text{TN}}}}_{{{\text{ERA}}5}} \left( {{\text{m}}_{{\text{v}}} } \right) - \overline{{{\text{TN}}}}_{{{\text{S}}5}} \left( {{\text{m}},{\text{lt}}} \right) $$

where $\overline{{{\text{PR}}}}_{{{\text{ERA}}5}}$, $\overline{{{\text{TX}}}}_{{{\text{ERA}}5}}$, and $\overline{{{\text{TN}}}}_{{{\text{ERA}}5}}$ are the multiannual mean of ERA5 precipitation and maximum and minimum temperatures, respectively, for the verification month m_v_ (${\text{m}}_{{\text{v}}} = {\text{m}} + {\text{lt}}$, circular over 12 months); and $\overline{{{\text{PR}}}}_{{{\text{S}}5}}$, $\overline{{{\text{TX}}}}_{{{\text{S}}5}}$, $\overline{{{\text{TN}}}}_{{{\text{S}}5}}$ are the multiannual and ensemble means of the forecasts.

For persistence forecasts, let us consider that the issue month is March (bearing in mind that the forecast is issued on the 1st day of the month), and the target predictor month is May. In this case, the forecast for May is the known predictor at the date of issue, which in this example is the ERA5 predictor in February (i.e., in March, the known climate anomalies are those from the previous month). The persistence forecast^28^ is used here as the baseline forecast, i.e., the a priori knowledge that the farmer holds in the field based on empirical observation. An example of the methodology for persistent forecasts may be found on Supplementary Material, Fig. S1. Let’s assume the user wants to know the yield forecast in the issue month M, and the model predictors are in months M-2, M + 2, and M + 3. Predictor in M-2 is in the past and is therefore known at issue month. In this case, the ERA5 value of the predictor is used. However, predictors in months M + 2 and M + 3 are in the future relative to the issue month and are still unknown. For these predictors the persistent anomaly is used, i.e., we assign the anomaly for months M + 2 and M + 3 as being equal to the anomaly that is observed at issue month (thus the term persistence). Since the anomaly of month M is only known at the end of the month (i.e., effectively in the beginning of M + 1), the known predictors when the issue month M starts are those from the previous month M-1. Hence, predictors in months M + 2 and M + 3 are issued with the values of M − 1, i.e., the persistence.

### Regression models for cereal yield production

Grid points of ERA5/SEAS5 that overlap Region 1 and Region 2 are selected, accounting for a total of 8 and 10 grid points, respectively (Supplementary Material, Fig. S2). Then, the spatial averages of PR_ERA5_, TX_ERA5_, TN_ERA5_, ${\text{PR}}_{{{\text{S}}5}}^{^{\prime}}$, ${\text{TX}}_{{{\text{S}}5}}^{^{\prime}}$, and ${\text{TN}}_{{{\text{S}}5}}^{^{\prime}}$ are computed for the two regions. With the aim of understanding how well ERA5 and forecasts compare for the three variables used in this study (PR, TX, TN), a month-by-month Pearson’s linear correlation ${\text{R}}$ is computed between time-series of weather variables from: (1) ERA5 and SEAS5 and (2) ERA5 and persistence. Standardized anomalies are computed for all variables by removing the interannual linear trend and dividing by the interannual standard deviation, transforming PR_ERA5_, TX_ERA5_, TN_ERA5_, ${\text{PR}}_{{{\text{S}}5}}^{^{\prime}}$, ${\text{TX}}_{{{\text{S}}5}}^{^{\prime}}$, and ${\text{TN}}_{{{\text{S}}5}}^{^{\prime}}$ into ${\text{PR}}_{{{\text{ERA}}5}}^{{\text{a}}}$, ${\text{TX}}_{{{\text{ERA}}5}}^{{\text{a}}}$, ${\text{TN}}_{{{\text{ERA}}5}}^{{\text{a}}}$, ${\text{PR}}_{{{\text{S}}5}}^{{{a^{\prime}}}}$, ${\text{TX}}_{{{\text{S}}5}}^{{{a^{\prime}}}}$, and ${\text{TN}}_{{{\text{S}}5}}^{{{a^{\prime}}}}$.

The methodology employed here to develop linear models to predict wheat and barley production is similar to that used in Bento et al.^22^. A forward stepwise regression algorithm was applied to select groups of statistically significant predictors for linear regression models (p-value to choose predictor was 0.05). The pool of predictors is organized from October to June, i.e., the growing season of the cereals, and consists of ERA5 standardized anomalies (${\text{PR}}_{{{\text{ERA}}5}}^{{\text{a}}}$, ${\text{TX}}_{{{\text{ERA}}5}}^{{\text{a}}}$, ${\text{TN}}_{{{\text{ERA}}5}}^{{\text{a}}}$), accounting for a total of 27 predictors. Hence, the first year is 1994 (starting in October 1993 and ending in June 1994), and the last is 2019 (staring in October 2018 and ending in June 2019), i.e., 26 growing season periods. Consequently, 4 models are derived, representative of the two cereals and the two regions. With the aim of avoiding overfitting, a leave-one-year-out cross-validation method is used^28^. The predicted and observed wheat and barley yields, with and without cross-validation, are then compared using Pearson's linear correlation coefficient R, ${\text{R}}^{2}$ adjusted to the number of predictors in the regression model ${\text{R}}_{{{\text{adj}}}}^{2}$, and the mean absolute error (MAE).

### Forecasting cereal yield production

The four linear models developed with ERA5 predictors are now used with forecasted predictors, i.e., instead of using ERA5 variables, SEAS5 and persistence forecasts are used (reminding that persistence is actually the ERA5 fields known at the issue date). Let’s assume the user is growing winter wheat in Spain. In April, the user wants to have an idea of how much wheat is expected to harvest in the summer. Thus, he may use dynamical forecasts to estimate predictors from April to summer, or he may assume a persistent climate anomaly (e.g., if the previous months have been dry, then the next months will also be dry). These forecasted climate anomalies are used in the model, which is the one developed with the “observational” datasets (here with ERA5), giving a prediction of winter wheat that could be harvested in summer. This is the reason why we developed the models with ERA5 and then applied the forecasts of predictors to these models. Due to the forecast horizon of 7 months (the first lead time is for the current month, and the last lead time is issued 6 months before the current month), the forecasts may start up to 6 months prior to the later in the season predictor of each model (e.g., if the later predictor is for the month of May, a forecast of yield can be produced starting in November; if the later predictor is in April, the forecasts can start in October). Considering that winter wheat and barley start growing after the winter dormancy, in late winter or early spring (January to March), and that the user would beneficiate of an early warning several months before the harvest (~ July), the forecasts start in January. If, in a given issue month, the predictors’ month is in the past, then the ERA5 values are used (e.g., since the considered starting issue month is January, all predictors in October, November, and December are past months and thus are assigned ERA5 values). This method is used for both SEAS5 and persistence forecasts.

The forecasted wheat and barley anomaly yields (using both SEAS5 and persistence) are then verified by comparison with the observed anomalies. Contingency tables are built to allow for the probabilistic (yes/no) forecast verification of a given event^28,29^. Here, an event is defined as one year having yield losses if it presents a yield anomaly below zero in that given year. To validate the forecasts, three verification metrics are used: the false alarm rate (FAR), hit rate (H), and Heidke skill score (HSS)^28^. The HSS measures the fractional improvement of the forecast over the standard forecast, and it is normalized by the total range of possible enhancement over the standard. The range of the HSS is -∞ to 1. Negative values indicate that the chance forecast is better, 0 means no skill, and a perfect forecast obtains a HSS of 1. Furthermore, for each issue month, the distribution of the differences between forecasted and observed yield anomalies is presented in the form of boxplots, and the respective root mean square error (RMSE) is displayed to allow for a quantitative overview of the results. With the objective of quantifying the added value of using dynamical over persistence forecasts, the percent difference (${\text{P}}_{{{\text{diff}}}}$) is estimated as:

7 $$ {\text{P}}_{{{\text{diff}}}} \left( {\text{\% }} \right) = \left[ {1 - \frac{{{\text{RMSE}}_{{{\text{S}}5}} }}{{{\text{RMSE}}_{{{\text{per}}}} }}} \right] \times 100 $$

where ${\text{RMSE}}_{{{\text{S}}5}}$ and ${\text{RMSE}}_{{{\text{per}}}}$ are the RMSE between forecasted (SEAS5 and persistence, respectively) and observed yield anomalies. Negative values of ${\text{P}}_{{{\text{diff}}}}$ indicate the added value (in percentage) of using the dynamical forecasts relative to persistence.

The continuous ranked probability score (CRPS)^28^ was also estimated to compare yields obtained with the dynamic and persistence forecasts. Here, the full 25-member ensemble of SEAS5 forecasts was compared with the yield observations. It is important to note that although the SEAS5 is a probabilistic forecast, persistence is deterministic. Thus, the CRPS of the persistence is the mean absolute error (MAE).

### Ethics declaration

The plant collection and use was in accordance with all the relevant guidelines.

## Results

**Figure 2:**
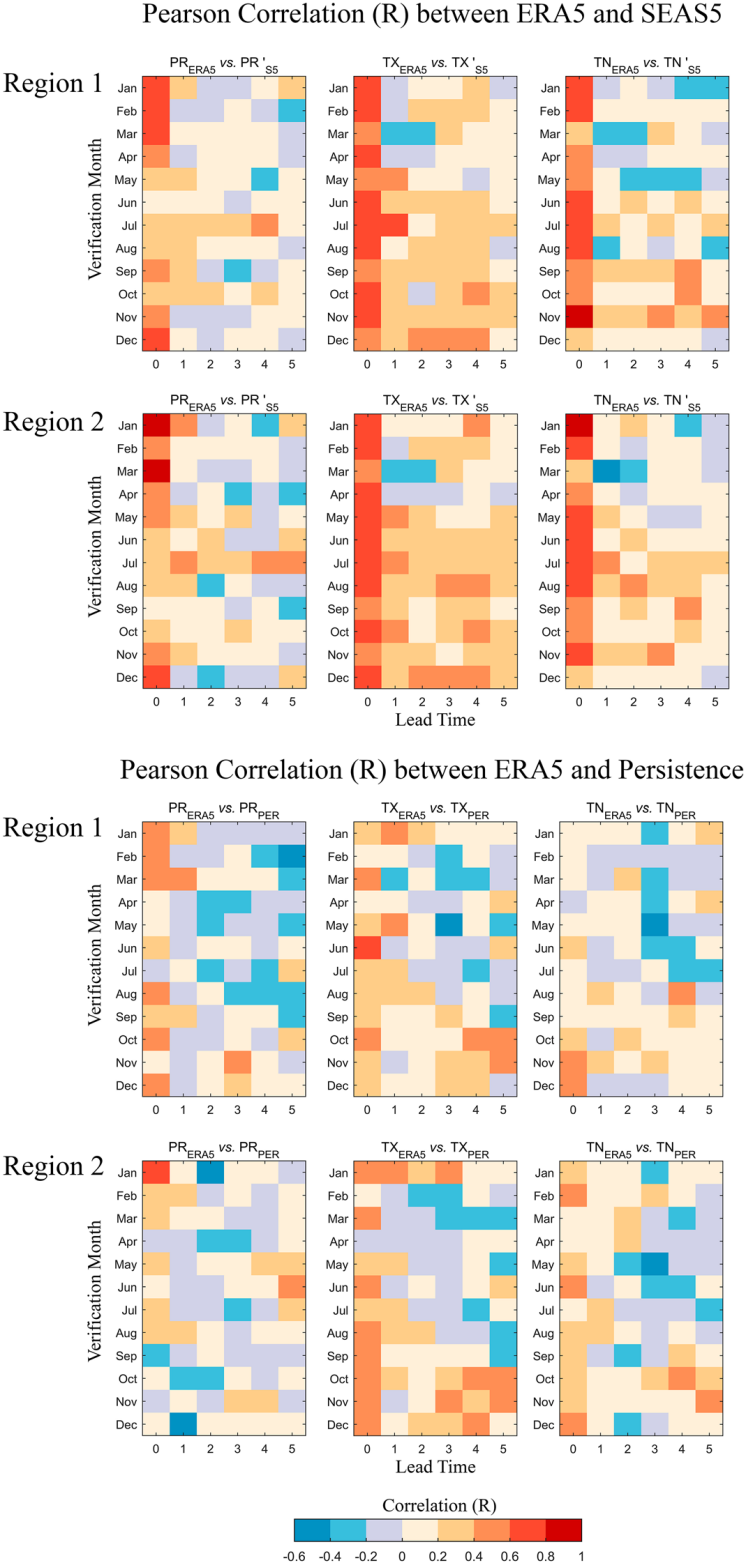
Pearson correlation coefficient (R) between monthly time series of PR, TX, and TN (left to right) from ERA5 and SEAS5 (top panels) and ERA5 and persistence (bottom panels). The figure was produced in Python 3.8 (https://www.python.org/).

The Pearson correlation between spatially averaged ERA5 and SEAS5 variables (1993–2019) for both regions is shown in Fig. 2 (top). As an example, precipitation in July with lead time 2 is the correlation between the SEAS5 forecast issued in May valid to July and ERA5 July precipitation. Differences between precipitation and temperature correlations are conspicuous. The former shows significant correlations essentially in the first month of forecasting (lead time 0, i.e., the issue month), except in late spring and summer, whereas both maximum and minimum temperatures show more frequent significant correlations, which tend to continue for longer lead times in some months.

A similar procedure is adopted for the persistence forecast, as shown in Fig. 2 (bottom). Using the same example as above, precipitation in July with lead time 2 is the correlation between ERA5 July precipitation and ERA5 April precipitation (in this case, the persistence is the value known when the forecast is issued two months in advance, on the 1st of May, i.e., ERA5 precipitation in April).

Then, the linear models for wheat and barley yield prediction are developed through a stepwise regression taking a pool of 27 potential predictors (${\text{PR}}_{{{\text{ERA}}5}}^{{\text{a}}}$, ${\text{TX}}_{{{\text{ERA}}5}}^{{\text{a}}}$, ${\text{TN}}_{{{\text{ERA}}5}}^{{\text{a}}}$ from October to June). Table 1 shows the resulting four models and the respective metrics with and without cross-validation (cv).

**Table 1:** Summary of linear models obtained through stepwise regression for wheat and barley yields (denoted $y$ in the equations) located in Region 1 and Region 2.

		Equation	R (R_cv_)	${\text{R}}_{{{\text{adj}}}}^{2}$ $\left( {{\text{R}}_{{{\text{adj}}.{\text{cv}}}}^{2} } \right)$	MAE (MAE_cv_)
Wheat	Region 1	${\text{y}} = - 0.34{\text{TX}}_{{{\text{Mar}}}}^{{\text{a}}} - 0.36{\text{TX}}_{{{\text{May}}}}^{{\text{a}}} + 0.53{\text{PR}}_{{{\text{Apr}}}}^{{\text{a}}}$	0.87 (0.82)	0.73 (0.62)	0.38 (0.45)
	Region 2	${\text{y}} = - 0.25{\text{TX}}_{{{\text{Dec}}}}^{{\text{a}}} + 0.33{\text{PR}}_{{{\text{Nov}}}}^{{\text{a}}} + 0.49{\text{PR}}_{{{\text{Mar}}}}^{{\text{a}}} + 0.69{\text{PR}}_{{{\text{Apr}}}}^{{\text{a}}}$	0.85 (0.78)	0.67 (0.52)	0.42 (0.52)
Barley	Region 1	${\text{y}} = - 0.39{\text{TX}}_{{{\text{Mar}}}}^{{\text{a}}} - 0.33{\text{TX}}_{{{\text{May}}}}^{{\text{a}}} + 0.53{\text{PR}}_{{{\text{Apr}}}}^{{\text{a}}}$	0.88 (0.82)	0.73 (0.63)	0.38 (0.45)
	Region 2	${\text{y}} = - 0.54{\text{TX}}_{{{\text{Nov}}}}^{{\text{a}}} - 0.36{\text{TX}}_{{{\text{Mar}}}}^{{\text{a}}} + 0.38{\text{TN}}_{{{\text{Oct}}}}^{{\text{a}}} + 0.33{\text{PR}}_{{{\text{Apr}}}}^{{\text{a}}} + 0.48{\text{PR}}_{{{\text{May}}}}^{{\text{a}}}$	0.91 (0.85)	0.78 (0.64)	0.33 (0.44)

The validation metrics considered are the Pearson correlation R, $R^{2}$ adjusted to the number of predictors in the regression model $R_{adj}^{2}$, and the mean absolute error (MAE) with and without leave-one out cross-validation (cv). The predictors are symbolized as PR^a^, TX^a^, and TN^a^ for precipitation, minimum and maximum temperature monthly standardized anomalies, respectively. The month in the subscript represents the month of the predictor.

Correlation coefficients between predicted and observed crop yields vary between 0.85 and 0.91 without cross-validation and show small signs of degradation when applying the leave-one out cross-validation scheme, ranging between 0.78 and 0.85. The model applied to barley in Region 2 has more predictors (five) and consequently has a better fit to the observed anomalies. Although the larger the number of predictors is, the ${\text{R}}^{2}$ adjusted to the number of predictors in the regression model ${\text{R}}_{{{\text{adj}}}}^{2}$ continues to be larger in this case, which establishes model robustness. The MAE varies between 0.33 (barley) and 0.42 (wheat) without cross-validation and between 0.44 and 0.52 with cross validation. Wheat and barley in Region 1 retained similar predictors focused into the spring months (March, April, and May), with small variations in the weights attributed. On the other hand, Region 2 (located more towards southern Spain) wheat and barley yield equations present a mix of winter and spring predictors. The only common predictor of the four cereal/region pairs is April precipitation, always with a positive contribution, i.e., favourable to larger cereal production. The maximum temperature in March was present in the three models and was absent in only wheat Region 2. In contrast to April precipitation, March maximum temperature has negative weights, i.e., higher maximum temperatures handicap the amount of wheat and barley produced. This agrees with the expected behaviour of the growing season cycles in this region^23^, where a good production year is defined by moderate precipitation and temperatures in early spring, substantial precipitation and below average temperatures in April and May, and a warm and dry June, which allows for a gradual maturation beginning the formation of filled grains^23,30^.

**Figure 3:**
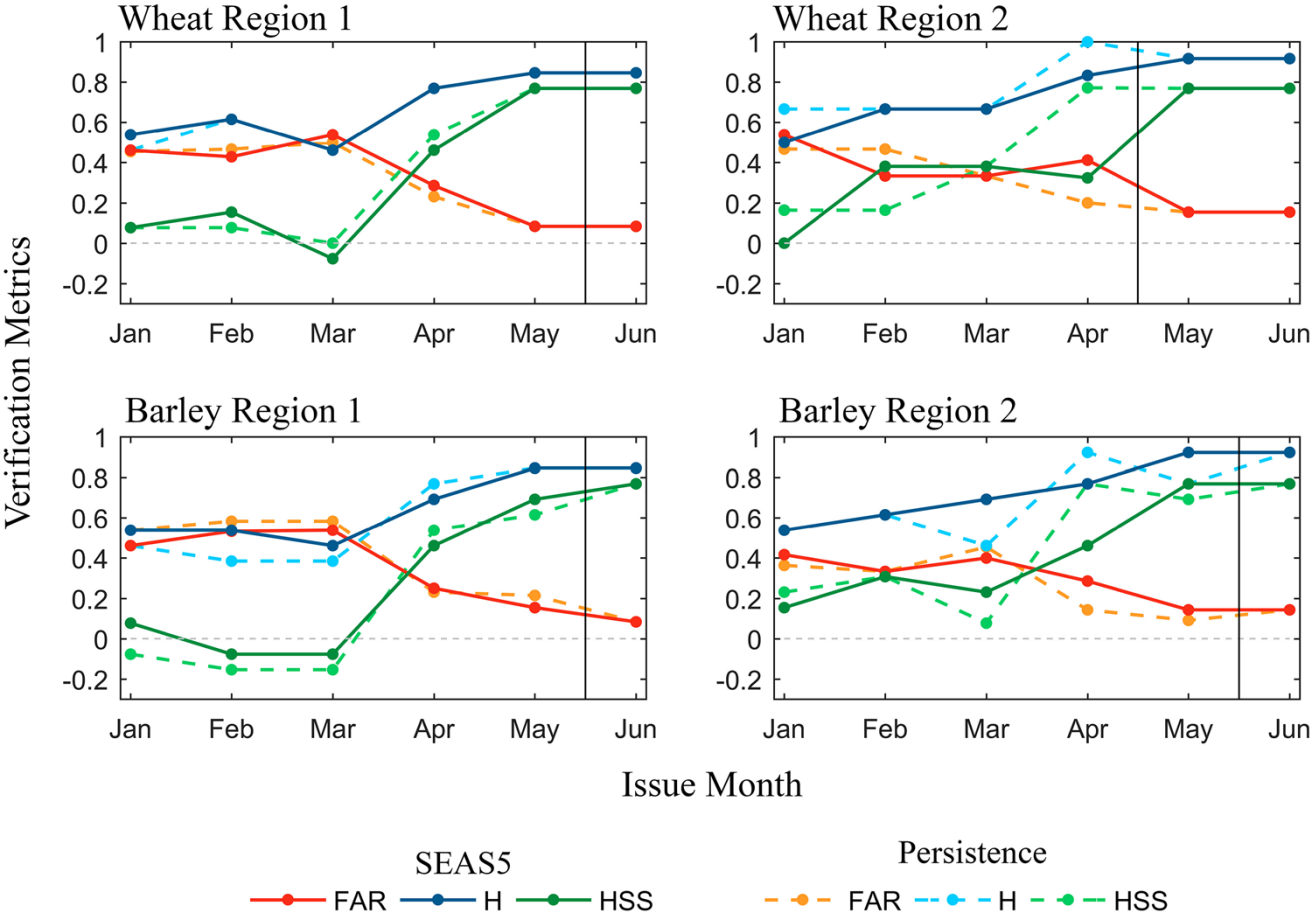
Forecast verification metrics: False alarm ratio FAR (orange), hit rate H (blue), and Heidke skill score HSS (green) for wheat yield anomalies (top) and barley yield anomalies (bottom) located in Region 1 (left) and Region 2 (right). Metrics for yields obtained with dynamical forecast SEAS5 are displayed in solid lines, whereas metrics for yields obtained with persistence forecasts are shown in dashed lines. The figure was produced in Python 3.8 (https://www.python.org/).

Linear models displayed in Table 1 are now evaluated with predictors from seasonal forecasts, the dynamical SEAS5, and the persistent climate. Figure 3 shows the verification metrics (FAR, H, and HSS) for the event of loss of yield (i.e., “yes” is yield anomaly < 0, and “no” is yield anomaly ≥ 0) starting in the issue month of January until the issue month of June. It is noteworthy that none of the models include predictors in June (see Table 1), which means that the verification metrics in June represent yield anomalies obtained using ERA5 predictors alone, thus being what we may call the “benchmark forecast” (separated by a vertical black line in Fig. 3). This benchmark forecast has some degree of error since correlations are not perfect, i.e., equal to 1 (see Table 1), and the verification metrics of Fig. 3 are not 1 (in the case of H and HSS) or 0 (in the case of FAR). Furthermore, the last predictor for wheat in Region 2 is April, which means that in addition to June, yield anomalies in May are also estimated with full ERA5 predictors (and consequently show identical results to verification metrics in June). Solid lines in Fig. 3 represent forecast verification metrics (H in blue; FAR in orange; HSS in green) for SEAS5, whereas dashed lines represent the same metrics for the persistence forecast. The results show no clear difference between SEAS5 and persistence. Indeed, the behaviour of both forecasts is similar, with larger differences occurring in Region 2. Notably, in this region, both wheat and barley yields forecasted in April show better skill when persistence is used (albeit showing lower accuracy, as discussed ahead). Furthermore, and as expected, these results show a tendency toward higher skill as the growing season progresses (and inclusion of more ERA5 predictors and subsequently fewer forecasted predictors). The results from Region 1 show low skill in the winter months for both SEAS5 and persistence and a significant leap in skill metric from April onwards (together with an increase in hit rate and a decrease in FAR).

**Figure 4:**
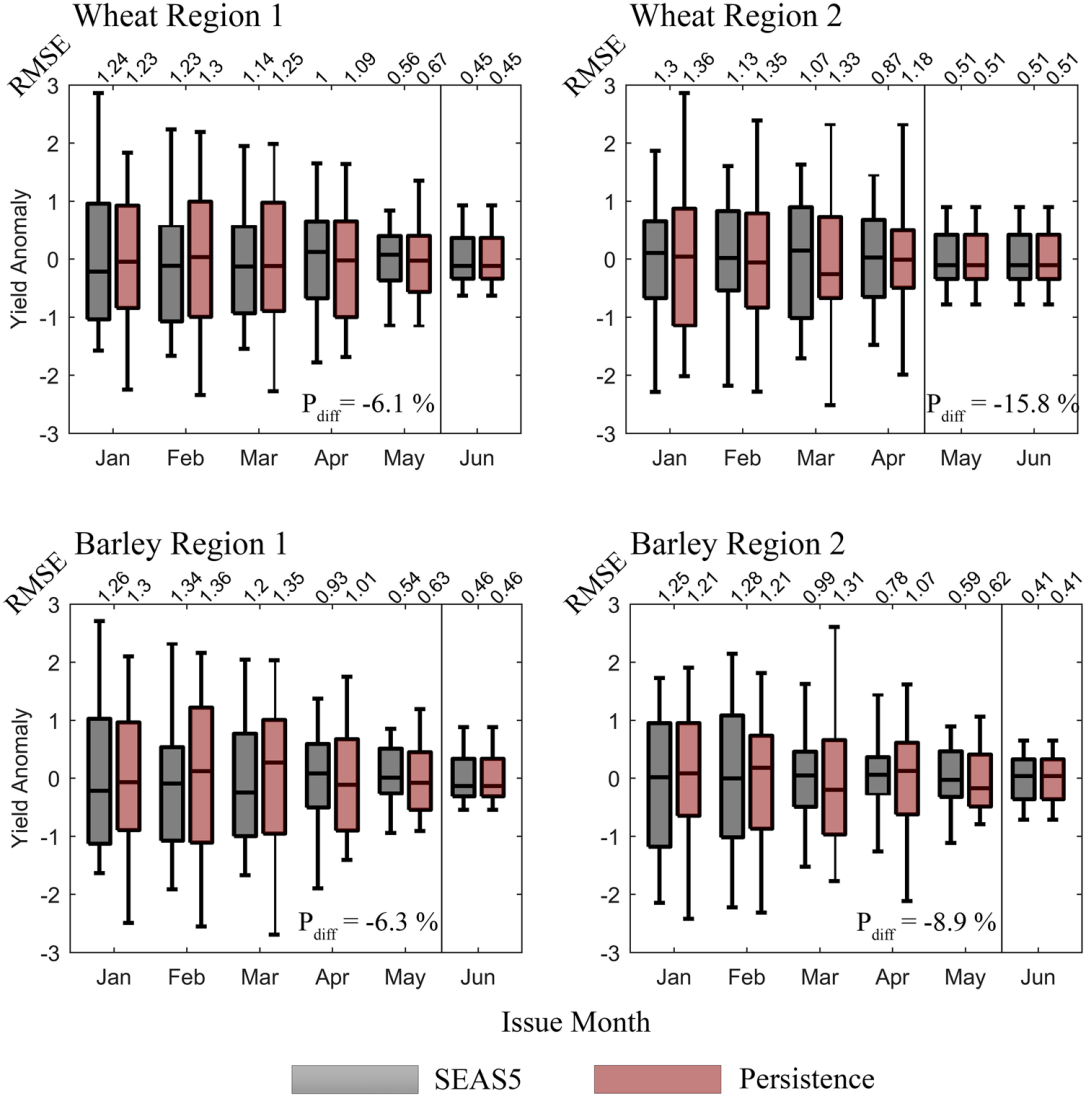
Distribution of differences between forecasted and observed yield anomalies of wheat (top) and barley (bottom) located in Region 1 (left) and Region 2 (right), encompassing the growing seasons of 1994–2019 (26 yields per issue month). Box plots filled in dark gray represent those differences estimated with the dynamical SEAS5 forecasts, whereas box plots filled in dark red represent differences when the forecast is assumed to be persistent. The x-axis represents the forecast issue month. Differences in anomalies of the “benchmark forecasts” are separated from the remaining anomalies by a vertical black line (June for wheat in Region 1 and barley in both regions and May and June for wheat in Region 2). On top of each box plot is displayed the RMSE, and inside each panel the percentage difference ${\text{P}}_{{{\text{diff}}}}$. The box ranges from the 25th to the 75th percentile, and the whisker ranges between the 5th and 95th percentiles. The median is illustrated as the horizontal black line inside the box. The figure was produced in Python 3.8 (https://www.python.org/).

The distribution of differences between forecasted and observed yield anomalies is shown in Fig. 4. Although similar results are displayed between SEAS5 and persistence (coherent with Fig. 3), some differences may be noted. As an example, for wheat and barley yield anomaly differences in Region 1 issued from February onwards, it is clear that there is a consistently larger interquartile distance when using persistence forecasts, meaning that the dynamical SEAS5 may be relatively more accurate than the persistence in forecasting yield anomalies of these cereals. The RMSE of forecasted versus observed yield anomaly supports this, with yield forecasts based on SEAS5 consistently having lower RMSEs than those related to persistence forecasts. Similar results may be found in Region 2 for barley yield anomalies. Here, differences between interquartile ranges are even larger, especially in the issue months of March and April (with RMSEs diverging by approximately 0.3). In the case of wheat in this region, the results show much more scattering from both forecasts and larger interquartile distances for the dynamical forecast in issue months of March and April. However, differences between persistent and observed wheat yield anomalies show larger extremes (whiskers tend to stretch to larger differences for both positive and negative values), which translates into more accurate forecasts when using the dynamical model (lower RMSE).

## Discussion

The rationale behind this research arises from the expected increase in socioeconomic pressure over the agricultural sector as a consequence of a fast-changing climate. Climate-related crop failures across multiple countries, namely, in Europe during 2003, 2010 and 2018^31–33^, have increased the need for adaptation to climate change. Therefore, reliable seasonal forecasts of crop yields are required for a timely response and to guideline stakeholders and policymakers. Particularly in the Iberian Peninsula, different spatiotemporal responses of wheat and barley to climate conditions have been suggested^18,21^, and our results provide further insight into the design of current crop monitoring and forecasting systems over these regions.

The results presented in this study demonstrate two different outcomes: (1) wheat and barley yield anomaly forecasts (dynamical and persistent) start to gain skill later in the season (typically from April onwards); and (2) the added value of using the SEAS5 forecast as an alternative to persistence ranges from 6 to 16%, with better results in the southern Spanish regions.

Relative to the first outcome, similar results were also found in^17^, indicating a lack of skill in the winter months and an increase in skill when using seasonal forecasts of SEAS5 issued later in the season in Spain for wheat flowering prediction, supporting our findings. Nevertheless, it is relevant to stress that the cited work and others focused on different regions of the world^13,16,34^ use information from process-based crop models forced with seasonal forecasts. Indeed, empirical models similar to those developed in this study^15,18,19,21,35^ do not integrate a comprehensive formulation of the physical processes that drive crop and climate interactions, intending only to represent large-scale impacts of climate on yields. However, empirical models are shown to typically be capable of reproducing results obtained from mechanistic models^36,37^, and due to their simplicity, they may be more friendly to be employed by stakeholders to set management strategies^7^. The usefulness of skilful forecasts issued from April onwards will depend on factors related to these strategies. Indeed, harvesting typically occurs in late spring/early summer, and this study indicates that stakeholders may start receiving skilful forecasts approximately one to two months ahead (typically in April/May). According to a survey done to U.S.A. winter wheat stakeholders, management decisions must take place 0–2.5 months before operationalization^38^, which is within the lead times discussed here. However, the extent to which complex dynamical seasonal forecasts introduce an added value when compared with forecasts based on the knowledge of the meteorological conditions that occurred in previous months is a relevant issue to be addressed. This leads to the discussion of the second outcome of this study.

The forecast verification metrics show that there is no major added value in using dynamical forecasts instead of persistence forecasts in terms of event configuration. Indeed, if the interest of the stakeholder is to merely know with a few months in advance if the yield of a given year will be better or worse than the mean yield of the previous decades, then using persistence is sufficient, as it seems to produce similar forecasting skill as using ECMWF SEAS5 as input. However, the same is not true if the stakeholder intends to have a more accurate forecast of the cereal yield. In this case, the results point to better achievable accuracy when employing the dynamical forecast system. Indeed, percent differences (${\text{P}}_{{{\text{diff}}}} )$ of RMSE obtained for dynamical forecasts versus observations relative to persistence forecasts versus observations (considering all lead times included) are always negative, ranging from about − 6% for both cereals in Region 1 to circa − 16% and − 9% for wheat and barley in Region 2, respectively. Similar results were obtained in another study^16^ for the Mediterranean region of Australia (with a similar climate to that of Iberia). There, the authors concluded that the dynamical forecasts had a narrower prediction range (more accurate) than the climatology driven ones (the authors used climatology forecasts instead of persistence), with the handicap of inducing more misleading forecasts than the climatology (lower skill scores). With the implementation of the probabilistic forecast score CRPS, results show an increase towards a larger added value in using SEAS5 instead of persistence (Supplementary Fig. S3). However, this metric is intended to evaluate probabilistic forecasts, and persistence is a deterministic forecast, which partially explains the larger differences obtained.

It is also noteworthy to highlight that the equation to estimate wheat in Region 2 presents three out of four predictors dependent on precipitation. This is a variable that presents small predictive capacity even for short lead times (see Fig. 2), a matter also discussed in^39^ and^40^. Furthermore, the models developed here (Table 1) are largely dependent on the size and quality of the databases used, and thus, more detailed wheat and barley production datasets would be ideal to increase the robustness of the results. Finally, the methodology employed here for wheat and barley may be applied to other crops with different outcomes. Future work may also focus on understanding how teleconnection patterns relevant for springtime influence the ability of persistent forecasts to achieve good forecasting skill. Indeed, both precipitation and temperature are influenced by patterns such as the North Atlantic Oscillation (NAO), the East Atlantic (EA) pattern, the El Niño–Southern Oscillation (ENSO), or the Mediterranean Oscillation (MO)^41–44^.

## Concluding remarks

Timely knowledge of yield before harvest is a critical piece of information that could help stakeholders take the necessary actions to maximize production and avoid expenses that may affect small farmers and country economies alike. This is where seasonal forecasts of meteorological variables play a crucial role. Indeed, permanent advances in dynamical forecasting systems, such as the state-of-the-art ECMWF SEAS5, may bring new possibilities in this field^45,46^. Here, this system is used to forecast wheat and barley yield anomalies taking advantage of multiple linear regression models and is further compared to what may be called the “poor-man” forecasting system of issuing forecasts based on persistent climate. Our results indicate that persistence is as good as the dynamical system in terms of predicting gains or losses of wheat and barley at different lead times, but the dynamical SEAS5 allows for a more accurate choice (6–16% RMSE improvement). These results, focused on crops located on the Iberian Peninsula, should provide guidelines on the design of crop monitoring and forecasting systems and give an idea to the regional stakeholder whether to invest or not in such forecasting techniques, depending on the objectives and requirements of the undertaking. Finally, it is important to acknowledge that the Iberian Peninsula is part of the Mediterranean basin, well known for being a major climate change “hot spot” where temperatures are rising faster than the world average while precipitation is slightly declining^47^. Thus, some of these key climate variables selected by our crop regression models for the two considered regions are not stationary. Not only have they been changing in recent decades but are bound to change even further in coming decades due to unavoidable warming scenarios (IPCC, 2021). To what extent this could undermine some of our results should be the focus of further research.

## Supplementary Information

Supplementary Information.

## Data Availability

Cereal production data is available at https://www.mapa.gob.es, and ERA5 and SEAS5 are available at https://cds.climate.copernicus.eu/.
