# Supplementary material for: Persistence versus dynamical seasonal forecasts of cereal crop yields
Source: Sci Rep. 2022 May 6;12:7422. doi: 10.1038/s41598-022-11228-2 (PMC9076871; doi:10.1038/s41598-022-11228-2)
Supplement: Supplementary file 1 — Supplementary Information. [file 41598_2022_11228_MOESM1_ESM.docx]

Persistence versus dynamical seasonal forecasts of cereal crop yields

Virgílio A. Bento^1,*^, Ana Russo^1^, Emanuel Dutra^1,2^, Andreia F.S. Ribeiro^3,1^, Célia M. Gouveia^1,2^ and Ricardo Trigo^1^

^1^ Instituto Dom Luiz, Faculdade de Ciências da Universidade de Lisboa, 1749 – 016 Lisboa, Portugal

^2^ Instituto Português do Mar e da Atmosfera, I.P., Rua C do Aeroporto, 1749 – 077 Lisboa, Portugal

^3^ Institute for Atmospheric and Climate Science, ETH Zurich, Universitätstrasse 16, Zurich 8092, Switzerland

Corresponding author: Virgílio A. Bento, Instituto Dom Luiz, Faculdade de Ciências da Universidade de Lisboa, 1749 – 016 Lisboa, Portugal. E-mail address: [vabento@fc.ul.pt](mailto:vabento@fc.ul.pt). ORCID: 0000-0001-9574-3090


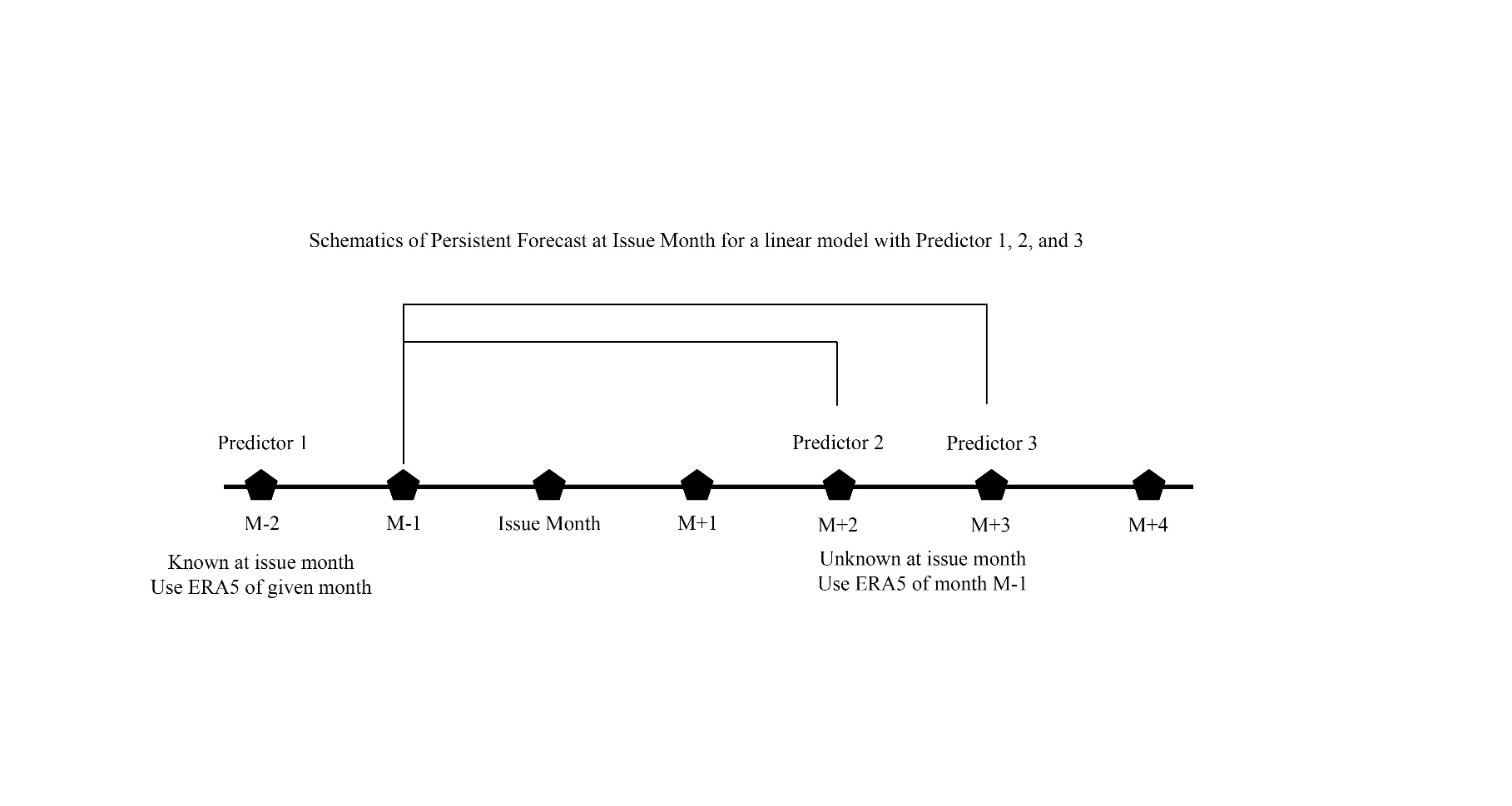


Figure S1 – Schematics of persistence forecast at a given issue month for a linear models fed with three predictors at months M-2, M+2, and M+3. At issue month, the value of predictor in M-2 is already known and ERA5 is therefore used. Conversely, at months M+2 and M+3 the persistent forecast is used as the value of the variable in the last known month (M-1). The figure was produced in Python 3.8 (https://www.python.org/).


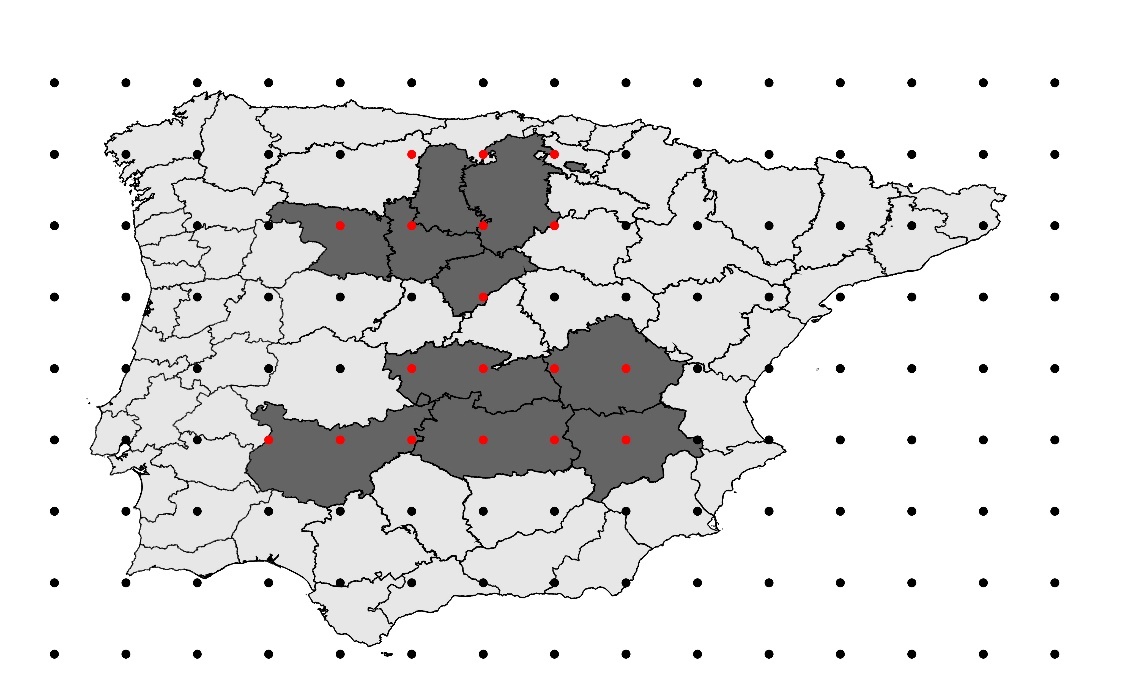


Figure S2 – Map overlapping the 1 x 1 grid from ECMWF with the map of provinces. Red dots represent the grid-points that were chosen in the study. The figure was produced in Python 3.8 (https://www.python.org/).


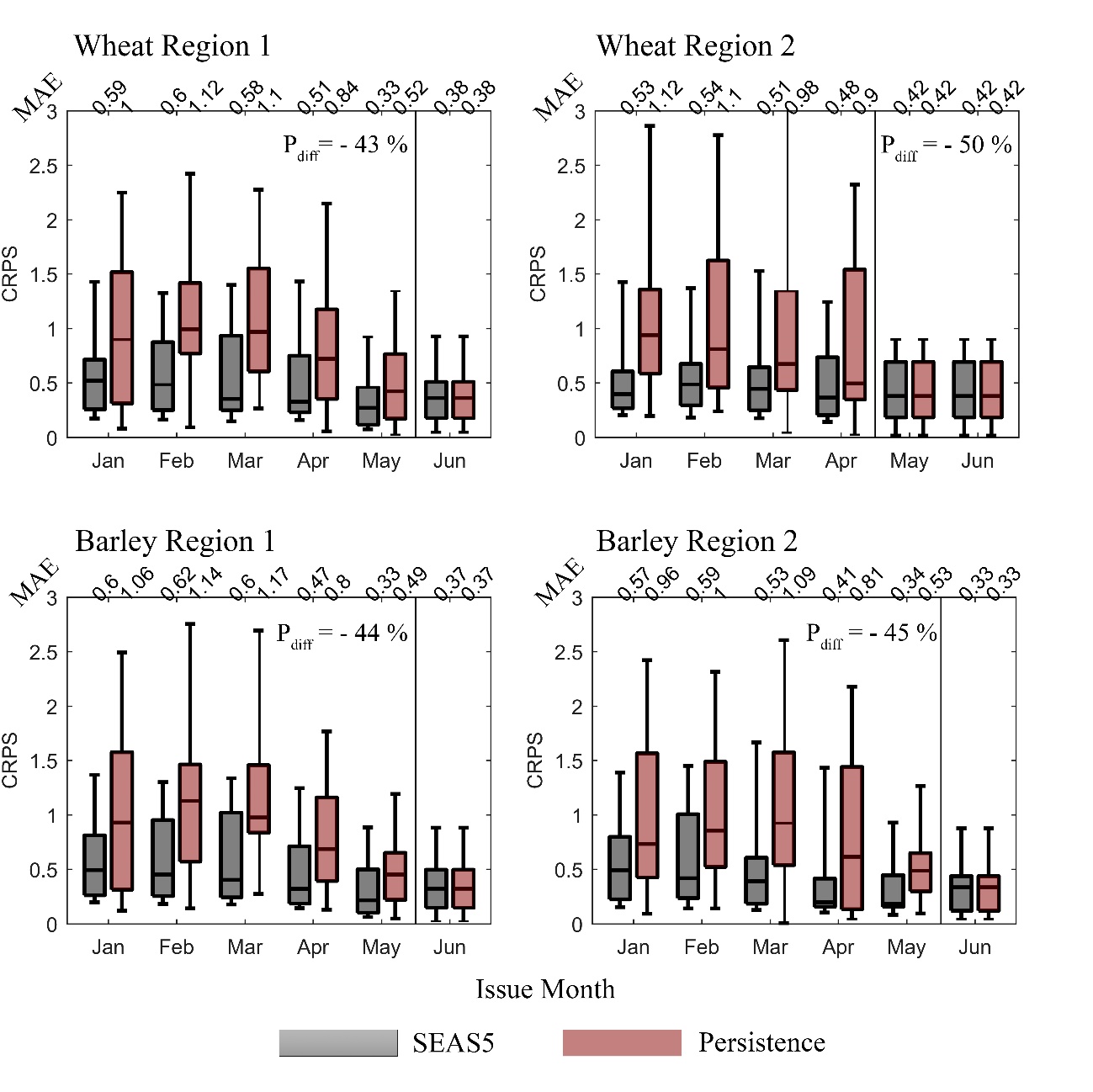


Figure S3 – The same as Figure 4 in the manuscript but using CRPS for the 25 ensemble members of the forecasts. The CRPS of the deterministic persistence is the absolute difference between persistent forecast and observation. RMSE was replaced by MAE to keep coherence with CRPS.
